# Plasma NfL, P‐Tau181 and and P‐Tau181/Aβ42 Ratio are associated with incident mild behavioural impairment in dementia‐free individuals

**DOI:** 10.1002/alz.095448

**Published:** 2025-01-09

**Authors:** Yingqi Liao, Joyce R Chong, Cheuk Ni Kan, Xuhao Zhao, Yuek Ling Chai, Mitchell Kim Peng Lai, Christopher Chen, Xin Xu

**Affiliations:** ^1^ Yong Loo Lin School of Medicine, National University of Singapore, Singapore Singapore; ^2^ Memory Aging and Cognition Center, National University Health System, Singapore Singapore; ^3^ School of Public Health, the Second Affiliated Hospital of School of Medicine, Zhejiang University, Hangzhou, Zhejiang China

## Abstract

**Background:**

Mild behavioural impairment (MBI) is associated with accelerated cognitive decline and conversion to dementia. However, the relationship between Alzheimer’s disease (AD)‐related pathological mechanisms and MBI remains unclear. This study aimed to examine whether baseline plasma neurofilament light chain (NfL), phosphorylated tau‐181 (P‐Tau181) and the P‐Tau181‐to‐Amyloid beta 42 (P‐Tau181/Aβ42) ratio are associated with incident MBI in an Asian cohort of dementia‐free individuals.

**Method:**

Dementia‐free participants were recruited from the community and memory clinics and underwent cognitive, neuropsychiatric and clinical assessments annually and brain MRI scans biennially at baseline and over a maximum of 5 years in an ongoing longitudinal study. Plasma NfL, P‐Tau181 and Aβ42 were measured using Single molecule array (Simoa) assays. Baseline MBI was defined as the presence of NPS at baseline and Y1 using the Neuropsychiatric Inventory, while incident MBI was defined as the development of new NPS persisting over any two consecutive years of follow‐up. The associations between baseline plasma biomarkers and incident MBI were examined using generalized estimating equation modelling. Additionally, competition risk survival analyses based on the Fine‐Gray model were performed to account for the competing risk of incident dementia. All models were adjusted for age, gender, education, clinical diagnosis (i.e. *No cognitive impairment (NCI) vs. Cognitively impaired‐No dementia (CIND)*) and the presence of cerebrovascular diseases (CeVD) at baseline.

**Result:**

Three hundred and five participants with complete NPI data and plasma biomarkers were included (age 72.1 ± 7.8 years, 52.5% female). Among the 248 without baseline MBI, 25.3% (n = 55) developed incident MBI. Elevated levels of plasma NfL (OR[95%CI] = 5.72(2.00, 16.36), p = 0.001) and P‐Tau181/Aβ42 ratio (OR[95%CI] = 3.54(1.03, 12.21), p = 0.05) were individually associated with incident MBI. Furthermore, when accounting for the presence of incident dementia and CeVD and plasma NfL, high levels of plasma P‐Tau181 were independently associated with an increased likelihood of incident MBI (HR[95% CI] = 2.44 (1.01, 5.86), p = 0.047).

**Conclusion:**

The relationship of plasma P‐Tau181, P‐Tau181/Aβ42 ratio and NfL with incident MBI suggests an association between AD‐pathology and neurodegeneration with late‐life behavioural changes. Therefore, combining clinical assessments with AD‐related plasma biomarkers may improve the early identification of at‐risk individuals for behavioural disturbances in dementia.

